# EEG biomarkers of activation of the lymphatic drainage system of the brain during sleep and opening of the blood-brain barrier

**DOI:** 10.1016/j.csbj.2022.12.019

**Published:** 2022-12-15

**Authors:** O.V. Semyachkina-Glushkovskaya, A.S. Karavaev, M.D. Prokhorov, A.E. Runnova, E.I. Borovkova, Ishbulatov Yu.M., A.N. Hramkov, D.D. Kulminskiy, N.I. Semenova, K.S. Sergeev, A.V. Slepnev, Sitnikova E. Yu., M.O. Zhuravlev, I.V. Fedosov, A.A. Shirokov, I.A. Blokhina, A.I. Dubrovski, A.V. Terskov, A.P. Khorovodov, V.B. Ageev, D.A. Elovenko, A.S. Evsukova, V.V. Adushkina, V.V. Telnova, D.E. Postnov, T.U. Penzel, J.G. Kurths

**Affiliations:** a Physics Department, Humboldt University, Newtonstrasse 15, 12489 Berlin, Germany; b Saratov State University, Astrakhanskaya str., 83, Saratov, 410012, Russia; c Charité – Universitätsmedizin Berlin, Charitéplatz 1, 10117 Berlin, Germany; d Saratov Branchof the Institute of Radio Engineering and Electronics of Russian Academy of Sciences, Zelyonaya, 38, Saratov, 410019, Russia; e Saratov State Medical University, B.Kazachaya str., 112, Saratov, 410012, Russia; f Institute of Higher Nervous Activity and Neurophysiology of Russian Academy of Sciences, (IHNA&NPh RAS), 5AButlerova St., Moscow 117485, Russia; g Institute of Biochemistry and Physiology of Plants and Microorganisms, Russian Academy of Sciences, ProspektEntuziastov13, Saratov 410049, Russia; h Potsdam Institute for Climate Impact Research, Telegrafenberg A31, 14473 Potsdam, Germany

**Keywords:** Lymphatic drainage system of the brain, Sleep, Opening of the blood-brain barrier, Electroencephalography, Non-linear analysis, Machine learning methods

## Abstract

The lymphatic drainage system of the brain (LDSB) is the removal of metabolites and wastes from its tissues. A dysfunction of LDSB is an important sign of aging, brain oncology, the Alzheimer's and Parkinson's diseases. The development of new strategies for diagnosis of LDSB injuries can improve prevention of age-related cerebral amyloid angiopathy, neurodegenerative and cerebrovascular diseases. There are two conditions, such as deep sleep and opening of the blood-brain-barrier (OBBB) associated with the LDSB activation. A promising candidate for measurement of LDSB could be electroencephalography (EEG). In this pilot study on rats, we tested the hypothesis, whether deep sleep and OBBB can be an informative platform for an effective extracting of information about the LDSB functions. Using the nonlinear analysis of EEG dynamics and machine learning technology, we discovered that the LDSB activation during OBBB and sleep is associated with similar changes in the EEG θ-activity. The OBBB causes the higher LDSB activation vs. sleep that is accompanied by specific changes in the low frequency EEG activity extracted by the power spectra analysis of the EEG dynamics combined with the coherence function. Thus, our findings demonstrate a link between neural activity associated with the LDSB activation during sleep and OBBB that is an important informative platform for extraction of the EEG-biomarkers of the LDSB activity. These results open new perspectives for the development of technology for the LDSB diagnostics that would open a novel era in the prognosis of brain diseases caused by the LDSB disorders, including OBBB.

## Graphical Abstract

**Figure:**
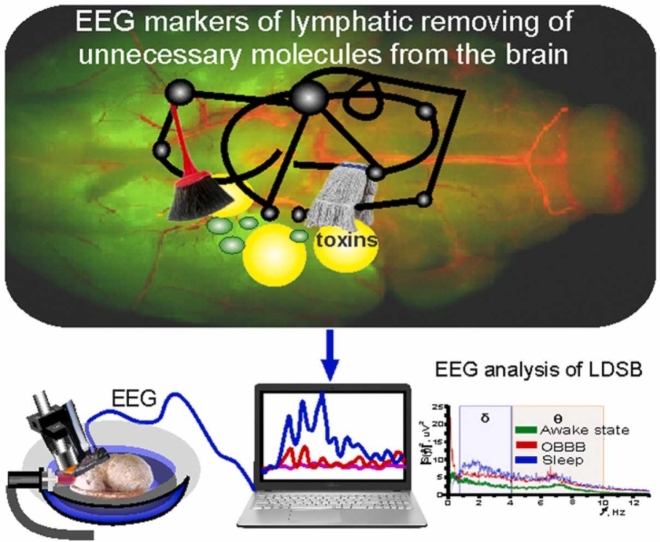

## 1 Introduction

The lymphatic drainage system of the brain (LDSB) is considered as a novel target for intervention of neurological diseases [1]. The LDSB plays a crucial role in maintenance of water and ion balance, clearance of metabolites and wastes as well as in communication with the immune system providing immune surveillance of the brain [2], [3], [4], [5], [6], [7], [8], [9], [10], [11]. A large number of brain diseases is associated with the LDSB impairment, including the Alzheimer's (AD) [6] and Parkinson's [12] diseases, brain tumors [13], [14], intracranial hemorrhages [7], [8], [9]. The dysfunction of LDSB is also an important characteristic of age-related changes of the brain [15]. Therefore, the development of new strategies for diagnosis of the LDSB injuries can significantly improve the prevention of age-related cerebral amyloid angiopathy, neurodegenerative and cerebrovascular diseases, and their complications. Furthermore, because of the importance of LDSB in immunity [4], [15], [16], the effective methods of assessment of the LDSB functions may enhance the therapeutic potential of immunotherapy for AD, neuroinflammation, brain tumors. More importantly, due to the unique functional roles of LDSB in drainage of the brain parenchyma, perspective technologies of monitoring of the LDSB functions may prove advantageous for brain tissue-targeted gene therapy and drug delivery. Given the immunological and pathophysiological significance of LDSB, it is thus valuable to consider development of prognostic technologies of LDSB that has the high clinical importance by taking advantage of the crucial role of LDSB in maintenance of the homeostasis of the central nervous system (CNS).

There are two unique conditions, such as deep sleep [17], [18], [19], [20] and opening of the blood-brain-barrier (OBBB) [11], [21], [22], [23], [24], [25], associated with the similar LDSB activation. For instance, both sleep [10], [18], [26] and OBBB [22], [27], [28], [29], [30] are accompanied by activation of clearance of beta-amyloid (Aβ) from the brain. During deep sleep is increased lymphatic clearance of wastes from CNS [18]. Similar scenarios are observed after OBBB. So, OBBB is associated with fast lymphatic clearance of unnecessary molecules penetrating in brain tissues via OBBB [11], [21], [22], [23], [24], [25]. Thus, sleep and OBBB can be an important informative platform for development of innovative methods of diagnosis of LDSB.

A promising candidate for measurement of LDSB during sleep and OBBB could be electroencephalography (EEG). Indeed, both deep sleep and OBBB are characterized by similar and certain changes in electrical activity of the brain in the form of low frequency of EEG dynamics [18], [19], [20], [31], [32]. There is hypothesis that the low frequency EEG pattern can be biomarker of the LDSB activation [17], [18], [19]. Slow cortical network oscillations (delta waves) may contribute to the efficiency of fluid influx into brain parenchyma and clearance of wastes from the brain [18], [33]. The reducing of low sleep activity is one of important diagnostic symptom of altered clearance of the brain associated with neurodegenerative diseases via accumulation of toxins in the brain [34], [35]. Delta waves are increased in naturally sleeping animals [18] and humans [20] as well as in subjects with OBBB [32], potentially due to long-term homeostatic changes in the neuromodulatory, metabolic, and neurochemical environment [36], broad synaptic scaling [37] and astrocytic mechanism of EEG modulation [38], [39], [40], [41], [42], [43].

We hypothesis that the special EEG changes during sleep and OBBB may be helping in “brain rinsing” and to move of brain fluids and waste products through brain tissues like sea waves move salt and water. Thus, sleep and OBBB can be an important informative platform for the extracting of EEG-biomarkers of the LDSB functions using the sensitive methods for the analysis of the EEG dynamics. To test this hypothesis, in this interdisciplinary study we used the original experimental design of real-time recording of the EEG patterns during OBBB and sleep with the non-linear analysis of the EEG dynamics and the machine learning technology.

## 2 Results

### 2.1 Deep sleep and OBBB are associated with the LDSB activation

In the first step of our study, we tested the hypothesis, whether deep sleep and OBBB can be an informative platform for an effective extracting information about the LDSB functions. To test our hypothesis, we created the functional model of in vivo study of LDSB in the OBBB, sleep and wake groups (Fig. 5). For in vivo experiments, the EEG signals recorded during 3 h after filling LDSB with tracer - the fluorescein isothiocyanate (FITC)-dextran 70 kDa (FTICD) injected into the right lateral ventricle and its lymphatic removing to the deep cervical lymph nodes (dcLNs) in the tested groups. This in vivo functional model was used for the further non-linear analysis of EEG characteristics of the LDSB activation. For each animal, a two channels cortical EEG/one channel electromyogram (EMG) were recorded. The silver electrodes were installed symmetrically in the left and right hemispheres (Fig. 5). Additionally, to confirm the in vivo data, the *ex vivo* experiments were carried out using confocal imaging of OBBB, distribution of FITCD in brain tissues and its accumulation in dcLNs.

**Fig. 1:**
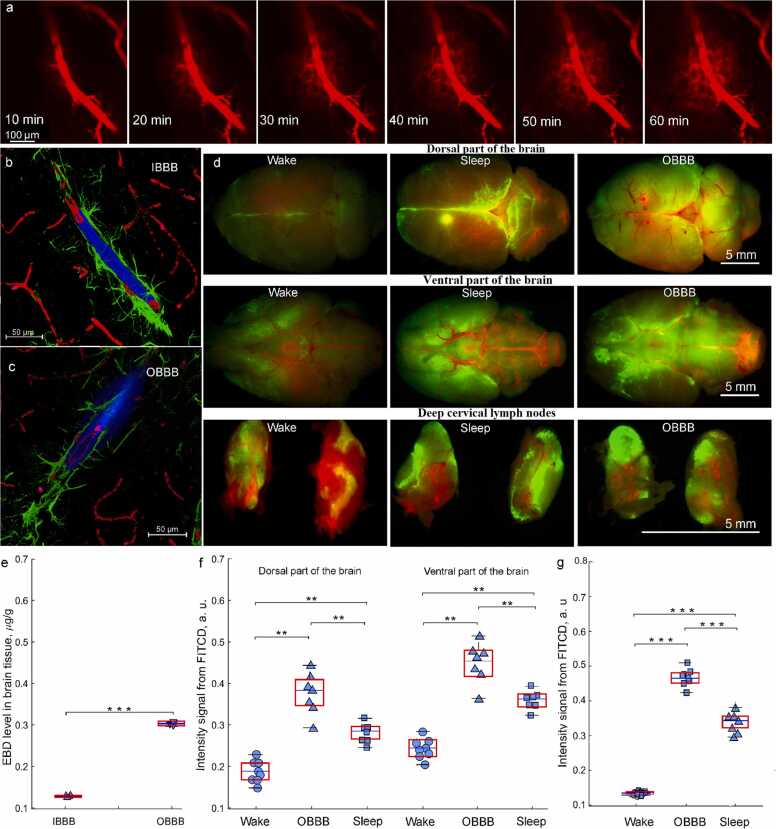
The LDSB activation during sleep and OBBB: a - the real time two-photon monitoring of music-induced OBBB to EBD (red) with an increase in dye leakage during 1 h in awake behaving rats with simultaneous EEG recording; b and c – the *ex vivo* confocal imaging of IBBB (b) and OBBB (c) to EBD using of the markers of pericytes (red) and astrocytes (green) demonstrating the EBD (blue) penetration behind the endothelial wall of the cerebral microvessels and its distribution between astrocytic end feet; d - the *ex vivo* confocal imaging of FITCD distribution from the right lateral ventricle in brain tissues and its accumulation in dcLNs in the wake, OBBB, and sleep groups 3 h after tracer intraventricular injection; e – the spectrofluorometric assay of the EBD level in brain tissues in rats with IBBB and OBBB, * ** - p < 0.001, the Mann–Whitney–Wilcoxon test, n = 7 in each group; f and g – the quantitative analysis of intensity of fluorescent signal from FITCD in the brain and in dcLNs in the tested groups, respectively, ***- p < 0.001, ** - p < 0.01, the Mann–Whitney–Wilcoxon test, n = 7 in each group.

Fig. 1a illustrates the real time two-photon monitoring of music-induced OBBB to the Evans blue dye (EBD) with an increase in dye leakage during 1 h in awake behaving rats with simultaneous EEG recording. Afterward, in *ex vivo* experiments, the qualitative and quantitative analysis of OBBB were performed to confirm OBBB to EBD. The spectrofluorimetric assay revealed the high EBD level in brain tissues of rats with OBBB vs. the intact BBB (IBBB) (0.33 ± 0.07 µg/g tissues vs. 0.12 ± 0.02 µg/g tissues, p < 0.001, the Mann–Whitney–Wilcoxon test, n = 7 in each group) (Fig. 1e). The confocal imaging of OBBB using the markers of pericytes and astrocytes demonstrated the EBD penetration behind the endothelial wall of the cerebral microvessels and its distribution between astrocytic end feet (Fig. 1b and c). Thus, our in vivo and *ex vivo* data clearly show than the EEG dynamics were recorded in awake behaving and unanesthetized rats with OBBB to EBD.

To analyze LDSB, we used our published protocol [10] based on the confocal imaging of FITCD distribution from the right lateral ventricle in brain tissues and its accumulation in dcLNs in the wake, OBBB, and sleep groups (Fig. 1d).

Both the sleep and OBBB groups vs. the wake group demonstrated the highest spreading of tracer that was more pronounced on the ventral than on the dorsal aspect of the brain 3 h after dye injection into the ventricle (0.48 ± 0.07 a.u. (OBBB) and 0.37 ± 0.05 a.u. (sleep) vs. 0.24 ± 0.03 a.u. (wake) on the ventral aspect of the brain; 0.36 ± 0.08 a.u. (OBBB) and 0.28 ± 0.04 a.u. (sleep) vs. 0.18 ± 0.01 a.u. (wake), p < 0.01 between the OBBB/sleep and the wake groups, the Mann–Whitney–Wilcoxon test, n = 7 in each group) (Fig. 1f). These data reflect the direction of FITCD movement from the ventricle to the basal meningeal lymphatic vessels located in the ventral part of the brain and playing an important role in the brain drainage and clearance [4], [5], [6], [7], [8], [9], [11], [15]. The dcLNs are the first anatomical station of CSF exit with dissolved unneeded substances for the brain [4], [5], [6], [7], [8], [9], [11], [15]. Therefore, we studied the accumulation of FITCD in dcLNs in three tested groups. Fig. 1d and g clearly show that the intensity from dye in dcLNs was higher in the sleep and OBBB groups compared with the wake group (0.47 ± 0.09 a.u. (OBBB) and 0.34 ± 0.05 a.u. (sleep) vs. 0.16 ± 0.06 a.u. (wake), p < 0.001 between the OBBB/sleep and wake groups, the Mann–Whitney–Wilcoxon test, n = 7 in each group).

Thus, our findings discovered the highest distribution of FITCD in brain tissues as well as its lymphatic removing into dcLNs in sleeping rats and with OBBB vs. awake animals. These data suggest the LDSB activation in the both sleep and OBBB groups. Our results are consistent with our previous data and other investigations showing activation of drainage and clearance of brain tissues during sleep and after OBBB in both animals [10], [11], [18], [19], [21], [22], [23], [24], [25], [32], [44], [45], [46] and humans [20], [27]. Notice that OBBB was accompanied by greater the LDSB activation than sleep (0.48 ± 0.07 a.u. vs. 0.37 ± 0.05 a.u., p < 0.01 in the ventral part of the brain; 0.36 ± 0.08 a.u. vs. 0.28 ± 0.04 a.u., p < 0.01 in the dorsal part of the brain; 0.47 ± 0.09 a.u. vs. 0.34 ± 0.05 a.u. in dcLNs, p < 0.001, the Mann–Whitney–Wilcoxon test, n = 7 in each group).

Thus, our data confirmed the hypothesis that sleep and OBBB vs. awake state are associated with the LDSB activation and can be an important informative platform for extracting the EEG biomarkers of the LDSB functions.

### 2.2 Non-linear EEG analysis of the LDSB activation during sleep and OBBB

**Fig. 2:**
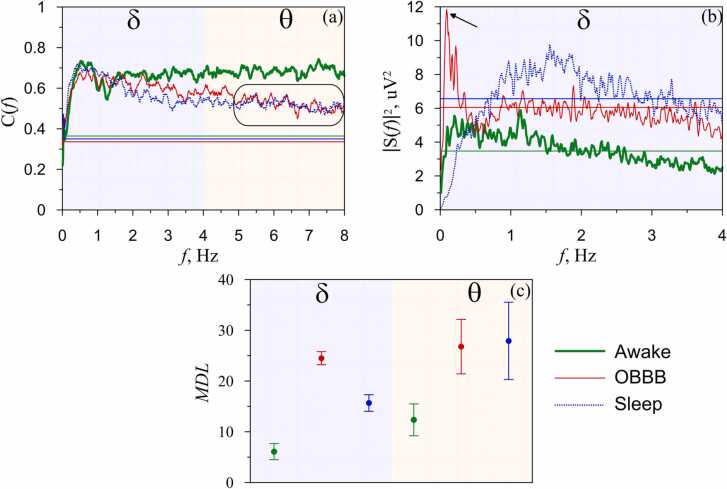
Non-linear analysis of the EEG dynamics in OBBB, sleep and wake groups: a – the coherence functions (the region of similar dynamics during sleep and OBBB is marked); b - the power spectra of EEG (arrow indicates the maximum value of the power spectrum of EEG during OBBB in the δ-band). The figure shows the spectra of signals recorded from lead 1 in the left hemisphere; c - CRA index *MDL*. The measures were calculated for each state and averaged over the statistical ensemble. In a and b horizontal lines correspond to the significance level (p < 0.05).

Using functional models of in vivo study of the LDSB activation, here we compared the EEG dynamics in the sleep, OBBB, and wake groups using the spectral analysis, estimates of the coherence function, and the maximum diagonal line (MDL) index calculated via the cross recurrence analysis (CRA). In the first step, we used the coherence function for the analysis of the EEG dynamics in three indicated conditions. The coherence function was calculated between a pair of EEG leads for each animal. Our results revealed that the EEG activity in the θ-rhythm (4–8 Hz) is very similar between sleep and OBBB (0.52 ± 0.01 and 0.53 ± 0.02, respectively, the Mann–Whitney–Wilcoxon test, n = 7 in each group) (Fig. 2a). However, both OBBB and sleep were significantly different from wakefulness (0.68 ± 0.01 (wakefulness) vs. 0.52 ± 0.01, p < 0.001 (sleep) and vs. 0.53 ± 0.02, p < 0.001 (OBBB), the Mann–Whitney–Wilcoxon test, n = 7 in each group). Thus, the coherence analysis of the EEG dynamics established that the OBBB-mediated θ-band in the EEG activity looks like sleep.

In the next step, using the power spectra, we analyzed the EEG characteristics in the low wave activity of δ-band (0.1–0.5 Hz) (Fig. 2b). Since the power spectra calculated from the signals of the EEG leads of the left and right hemispheres of each animal was similar, we presented in Fig. 2b the spectra of signals recorded from only one lead in the left hemisphere. The OBBB group was accompanied by a significant increase in the power spectrum that was less pronounced in the sleep and wake groups (11.85 ± 0.25 (OBBB) vs. 3.84 ± 0.02, p < 0.001 (sleep) and vs. 4.91 ± 0.06, p < 0.001 (awake state) the Mann–Whitney–Wilcoxon test, n = 7 in each group). Thus, these results clearly demonstrate that the low frequency EEG activity (0.1 Hz) reflects the EEG dynamics typical for OBBB.

Application of different nonlinear coupling detection techniques increases the reliability of our results and provides independent additional information. The CRA is a well-known nonlinear method to measure similarity of two complex systems dynamics. In this study, CRA was applied to the pairs of the EEG signals to analyze the data in a broader frequency band in comparison to the phase analysis, which has a history of successful application to complex experimental systems and signals [47], including the EEG signals [48], [49]. Reconstruction of phase space is an important stage of the CRA. We followed Taken’s recommendations [50] and used the delay method. As a time delay we used the minimum of the autocorrelation function. The dimension of the phase space was estimated using the false neighbor approach. A detailed description of the reconstruction procedures is provided in Supplementary information (SI).

The calculated values of the *MDL* index were presented in Fig. 2c. The results clearly demonstrate that in the θ-band the *MDL* index for sleep is very close to the *MDL* index for OBBB (27.95 ± 7.62 and 26.82 ± 5.37, respectively, the Mann–Whitney–Wilcoxon test, n = 7 in each group). Both sleep and OBBB were different from wakefulness (12.39 ± 3.14 (wakefulness) vs. 27.95 ± 7.62 (sleep), p < 0.05 and vs. 26.82 ± 5.37 (OBBB), p < 0.05, the Mann–Whitney–Wilcoxon test, n = 7 in each group).

Furthermore, the *MDL* index in the δ-band also was effective for extracting information about the LDSB activation during OBBB. Indeed, the *MDL* index for the δ-rhythm revealed significant differences between the three states and allowed us to clearly identify the LDSB activation during OBBB, which has a higher *MDL* index compared with sleep and awake state (24.52 ± 1.29 (OBBB) vs. 15.67 ± 1.63 (sleep), p < 0.05 and 6.10 ± 1.58, p < 0.01 (awake state), the Mann–Whitney–Wilcoxon test, n = 7 in each group). The calculation of the addition CRA indices are presented in SI.

Thus, the results presenting the *MDL* index are in good accordance with the spectrum and the coherence analysis of the EEG dynamics demonstrating two EEG markers of the LDSB activation, such as the EEG θ-activity that is similar for both sleep and OBBB and specific changes in the low EEG activity in δ-band (0.1 Hz) that is typical only for OBBB.

### 2.3 Wavelet analysis of the EEG markers of the LDSB activation during sleep and OBBB

**Fig. 3:**
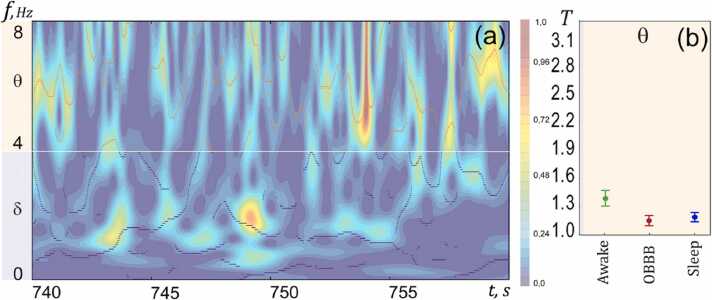
Analysis of the EEG oscillatory patterns based on continuous wavelet transform in the OBBB, sleep and wake groups: (a) – illustration of a typical wavelet spectrum *W* on the surface of frequency *f* and time *t*. The surface color corresponds to the power of the wavelet spectrum; (b) – statistical assessment of the duration *T* of oscillatory patterns in awake state, during sleep, and OBBB.

For a more detailed study of the EEG markers of the LDSB activity associated with OBBB and sleep, in the next step, we estimated the oscillatory patterns in the EEG dynamics in the OBBB, sleep and wake groups. Since in the previous step of experiments, we revealed similarity in the EEG θ-dynamics between OBBB and sleep, we used the wavelet surfaces *W* (*f*, *t*) and calculated the duration *T* of the existence of oscillatory patterns in the θ frequency ranges. A detailed description of estimation of these characteristics is provided in SI. The schematic process of the patterns detection is shown in Fig. 3a.

The duration *T* of oscillatory patterns for the three states (OBBB, sleep, and awake) in the θ-band was calculated for each rat. Afterward, we carried out a statistical analysis of the obtained characteristics in each group (Fig. 3b). The analysis of the duration *T* demonstrated a similarity for sleep and OBBB in the θ-band of oscillatory activity (1.18 ± 0.12 vs. 1.16 ± 0.12, respectively, the Mann–Whitney–Wilcoxon test, n = 7 in each group) and significant differences between awake state and sleep/OBBB (*T*_AWAKE_ = 1.47 ± 0.08 vs *T*_SLEEP_ = 1.18 ± 0.02, p < 0.001 and *T*_OBBB_=1.16 ± 0.02, p < 0.001, the Mann–Whitney–Wilcoxon test, n = 7 in each group).

Thus, the results of the wavelet analysis of the EEG dynamics are in a good agreement with the coherence and the spectrum analysis of the EEG patterns demonstrating typical for both OBBB and sleep θ-band in the EEG activity.

### 2.4 Machine learning analysis of the LDSB activity during sleep and OBBB

**Fig. 4:**
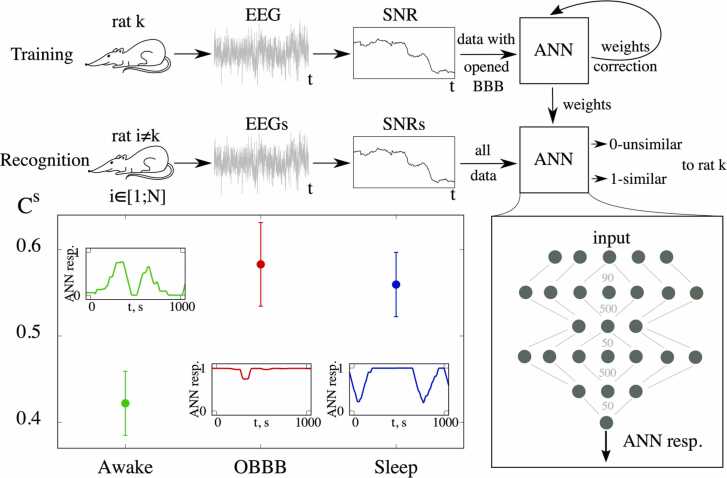
Machine learning analysis of the LDSB activity during sleep and OBBB: top and right panels – the application scheme of ANN; left panel – the part of EEG fragments recognized by ANN as corresponding to OBBB $C^{s}$ with plotted standard error of the mean for three cases: awake state, sleep, and OBBB. The subplots inside this panel show the examples of recognition of EEG fragments corresponding to the same cases.

In the final step, we analyzed the EEG dynamics in the OBBB, sleep and wake groups using artificial neural network (ANN) as a specific technique of machine learning. The scheme of this method is shown at the top of Fig. 4. The signal-to-noise ratio (SNR) calculated on the basis of EEG of rats was used as an input signal of ANN. The ANN was trained with a supervised learning. This technique requires the use of previously marked-up data, and for this purpose we used implementations with OBBB and natural behavior. The training data was obtained from an EEG of the 1st rat, and then the trained ANN was applied to the others. Then to train the another ANN the training data were taken from the EEG of the 2nd rat, etc. In this way, for the training data from each rat we trained a 6-layer ANN. The output layer had a single neuron, producing a real number in the range [0; 1], where 0 corresponded to maximum dissimilarity to training examples of OBBB, while 1 matched maximum similarity. Further this output value we called as a the network response. Then the value *C*^*S*^ was calculated to quantitatively compare the responses for OBBB, wakefulness, and sleep. This value showed the part of EEG fragments recognized by ANN as similar to EEG fragments from the training set. The structure of ANN and the method of *C*^*S*^ calculation is described in detail in Section “Analysis of EEG dynamics using an artificial neural network” in Materials and Methods. In the OBBB data, the averaged *C*^*S*^ was 0.58 ± 0.05 (Fig. 4). Using the same design of ANN, we obtained that the part of EEG realizations of normally sleeping rats was similar to training examples according to averaged *C*^*S*^= 0.56 ± 0.04, while for the wake group this value was 0.42 ± 0.04. Therefore, comparing the EEG dynamics in the OBBB, sleep and wake groups revealed that the similarity between sleep and OBBB was on 33.3 % higher than for awake state. These results clearly demonstrate that the EEG dynamics during OBBB is very similar to sleep that is consistent with our data obtained using the coherence, CRA, and wavelet analysis of the EEG activity.

## 3 Discussion

Our pilot studies on the healthy rats for the first time demonstrate that sleep and OBBB are the important informative platform for an effective extracting the EEG markers of the LDSB activity. Using the wavelets and the power spectra analysis of the EEG dynamics combined with the coherence function, we discovered that the LDSB activation during OBBB and sleep is associated with similar changes in the EEG θ-activity. The methods of artificial neural network as a technique of machine learning confirmed the similarity of the EEG dynamics during OBBB and sleep. The higher LDSB activation during OBBB vs. sleep is accompanied by specific changes in the low frequency EEG activity (0.1 Hz). Thus, sleep and OBBB are two unique conditions associated with the LDSB activation, which have similar changes in the EEG θ-activity, while more pronounced the LDSB activation during OBBB vs. sleep is accompanied by special changes in the EEG pattern in the form of the low frequency EEG dynamics.

The interrelation between sleep and OBBB can be explained by activation of clearance of macromolecules, metabolites, and toxins from the brain that is typical for both states. Indeed, sleep [10], [18], [26] and OBBB [22], [27], [28], [29], [30] are accompanied by clearance of Aβ from the brain. The sleep is characterized by coupled oscillations of the neural activity and CSF, which cleans metabolic wastes from the brain [20]. In clinical and experimental works have been shown that OBBB is associated with both activation of clearance of macromolecules from the brain [11], [21], [22], [23], [27] and with the changes in the EEG dynamics [22], [31], [32], [44], [45], [46].

We suppose that the clearance of different compounds from the brain can be a possible bridge between similar changes in the EEG dynamics co-occurring during sleep and OBBB. It is believed that an increase in the volume of the interstitial fluid (ISF) contributes by drainage of water-soluble metabolites from ISF to CSF compartments [51]. The sleep is associated with an increase in the volume of the interstitial space [18], [52] that is accompanied by an activation of macromolecular diffusion in the brain tissues [18]. The astrocytes rapidly and significantly change their volume, making a decisive contribution to the change in the total proportion of volume of ISF [53], [54], [55]. These dynamic astrocyte volume changes may represent a previously unappreciated yet fundamental mechanism by which astrocytes regulate brain rhythm during sleep [56], [57] and the BBB integrity [58], [59].

Other important aspect of our data is using the functional model of OBBB-mediated LDSB activation that allowed us to extract the important information about the specific EEG changes in form of the low frequency EEG oscillations associated with OBBB. Our findings are also in line with animal data of others demonstrating that OBBB by injection of mannitol [31] or by heavy stress [60] leads to the specific EEG changes.

The mechanisms of OBBB-mediated changes of neural activity is not fully understood. The OBBB can affect the EEG activity by direct and indirect ways. A direct influence of OBBB on the EEG dynamics is the generation of signals via electrophysiological properties of brain endothelial cells forming the BBB. These signals are originated from a trans-endothelial voltage between blood and brain tissue [61], [62], [63]. This voltage is a consequence of unequal endothelial cell apical and basolateral membrane potentials [64]. The ion influx/efflux affects OBBB via brain endothelial cells membrane depolarization affecting the cell stiffness via molecular mechanisms underlying cortical actin cytoskeleton [65], [66]. These changes of cell potential cause up to mV-level shifts in human scalp EEG [67]. Kiviniemi et al. observed that the intact BBB maintains the positive voltage, while the BBB leakage is characterized by a negative shift in this parameter [31].

Already in the 1970′s, it was discovered that the large-amplitude brain-potential shifts originate from a potential difference, which can occur during OBBB induced by respiratory acidosis in different animals species, including cats, monkeys, and rats [61], [62], [67]. On the one hand, there is evidence suggesting that BBB acts as a non-neuronal signal generator of mV-level slow shifts measured at scalp [66]. On the other hand, the BBB signals can also be coupled to neuronal function, since low level frequency oscillations in human brain are synchronized with faster cortical EEG oscillations and they are associated with the slow fluctuations in brain excitability [68], [69], [70].

Indirect influences of BBB on the EEG behavior are the astrocytes [71], which are essential for the formation and maintenance of BBB. Reduction in astrocyte number in the mPFC was associated with impaired cognitive flexibility and reduced power across delta (1–4 Hz), alpha (12–20 Hz), and gamma (30–80 Hz) frequency ranges [72], [73]. The astrocytic mechanism of EEG modulation can be mediated via astrocytes-mediated regulation of the synaptic conductance [74], [75], [76], which are involved in electrically induced EEG-activated states in cortical neurons [77].

## 4 Conclusion

Taking together, our pilot study on healthy rats demonstrate a link between neural activity associated with the LDSB activation during sleep and OBBB. Using the nonlinear analysis of the EEG dynamics, we discovered that the LDSB activation during OBBB and sleep is associated with similar changes in the EEG θ-activity. The machine learning technique, namely as ANN, confirmed the similarity of EEG activity during OBBB and sleep. The OBBB causes higher the LDSB activation vs. sleep that is accompanied by specific changes in the low frequency EEG activity (0.1 Hz) extracted by the power spectra analysis of the EEG dynamics combined with the coherence function.

Thus, our findings clearly demonstrate that sleep and OBBB are an important informative platform for extraction of the EEG-biomarkers of the LDSB activity. These results open the new perspectives for the development of technology for assessment of the LDSB functions that is of highest clinical importance and would open a novel era in the prognosis of brain diseases that causes the LDSB disorders, including OBBB.

Our findings have the some limitations. We used only functional model of the LDSB activation, such as sleep and reversible OBBB without brain pathology that limits the information about the LDSB dysfunctions related to brain diseases. Never OBBB and sleep were analyzed using combination of the non-linear analysis and machine learning technology, such as ANN that limits comparison of other approaches and their effectiveness. This needs further detailed human and animal studies of the LDSB functions in both physiological and pathophysiological conditions.

## 5 Material and methods

### 5.1 Subjects

The experiments were conducted on three groups of adult male Wistar rats (250–280 g or 8–10 week old) in: (I) awake state; (II) sleep; (III) OBBB, n = 7 in each group in all series of experiments. All procedures were performed in accordance with the “Guide for the Care and Use of Laboratory Animals”. The experimental protocols were approved by the Local Bioethics Commission of the Humboldt University and the Saratov State University. The experiments were done in accordance with the “Guide for the Care and Use of Laboratory Animals” [78]. The animals were kept in a light/dark environment with the lights on from 8:00–20:00 and fed ad libitum with standard rodent food and water. The ambient temperature and humidity were maintained at 24.5 ± 0.5 °C and 40–60 %, respectively.

#### 5.1.1 The LDSB analysis in the OBBB, sleep and wake groups

**Fig. 5:**
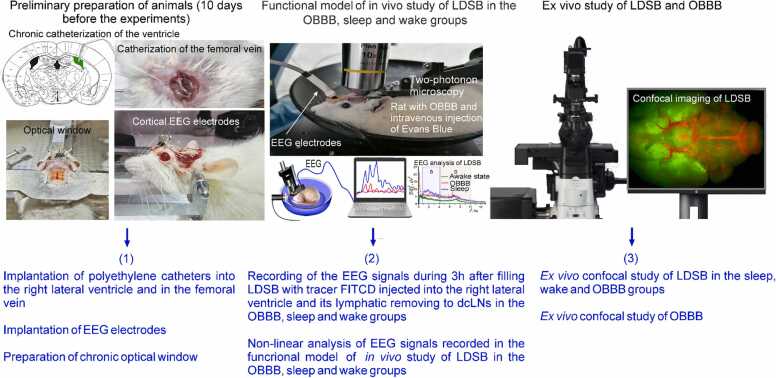
Design of in vivo and *ex vivo* experiments of study of LDSB in the OBBB, sleep and wake groups.

Fig. 5 illustrates of a design of in vivo and *ex vivo* experiments of study of LDSB in the OBBB, sleep and wake groups:

##### 5.1.1.1 Preliminary preparation of animals

(1) ten days before experiments, surgical procedures were performed, including (i) implantation of chronic polyethylene catheters into the right lateral ventricle (AP, 1.0 mm; ML, - 1.4 mm; DV, 3.5 mm) for the injection of FITCD (5 µl, at a rate of 0.1 µl/min, 0.5 % solution in saline, Sigma-Aldrich, US) and in the femoral vein for the Evans Blue dye injection; (ii) implantation of the EEG electrodes into the cortex; (iii) preparation of the chronic cranial window;

##### 5.1.1.2 Functional models of in vivo study of LDSB

(2) for in vivo experiments, the EEG signals recorded during 3 h after filling LDSB with FITCD injected into the right lateral ventricle and its lymphatic removing to dcLNs in the OBBB, sleep and wake groups. This in vivo functional model was used for the further non-linear analysis of the EEG characteristics of the LDSB activation;

##### 5.1.1.3 Ex vivo study of LDSB and OBBB

(3) *Ex vivo* study of LDSB was carried out using confocal imaging of FITCD spreading from the right lateral ventricle in the brain tissues and its accumulation in dcLNs in the OBBB, sleep and wake rats. The confocal imaging of OBBB to EBD using the markers of astrocytes and pericytes as well as spectrofluorometric assay of dye level in the brain tissues were performed.

### 5.2 EEG and EMG recording

A two-channels cortical EEG/one EMG (Pinnacle Technology, Taiwan) were recorded. The rats were implanted two silver electrodes (tip diameter 2–3 µm) located at a depth of 150 µm in coordinates (L: 2.5 mm and D: 2 mm) from Bregma on either side of the midline under inhalation anesthesia with 1 % isoflurane at 1 L/min N_2_O/O_2_ – 70:30. The head plate was mounted and small burr holes were drilled. Afterward, EEG wire leads were then inserted into the burr holes on one side of the midline between the skull and underlying dura. EEG leads were secured with dental acrylic. An EMG lead was inserted in the neck muscle. Ibuprofen (15 mg/kg) for the relief of postoperative pain was provided in their water supply for two to three days prior to surgery and for three or more days post-surgery. The rats were allowed 10 days to recover from surgery prior to beginning the experiment.

Since the standard sleep staging rules for rats are not available currently, we referred the visual scoring criteria from these studies [18], [19]. Wakefulness, non-rapid eye movement (NREM), and rapid eye movement (REM) were visually scored in 10 s epochs. EEG activity was measured and compared in rats in awake state, during sleep, and after OBBB on the same rats. Wakefulness was defined as a desynchronized EEG with low amplitude and high-frequency dynamics (>10 %, 8–12 Hz) and relatively high-amplitude EMG. NREM sleep was recognized as synchronized activity with high amplitude, which is dominated by low-frequency delta waves (0–4 Hz) comprising> 30 % of EEG waveforms/epoch and a lower amplitude EMG. REM was identified by the presence of theta waves (5–10 Hz) comprising> 20 % of EEG waveforms/epoch with a low EMG amplitude.

### 5.3 Music-induced OBBB

The detailed description of protocol of music-induced OBBB is described in Ref. [11], [21], [22], [25]. To produce the music (70–90–100 dB and 11–10000 Hz, Scorpions “Still loving you”) we used loudspeaker (ranging of sound intensity 0–130 dB, frequencies 63–15000 Hz; 100 V, Yerasov Music Corporation, Saint Petersburg, Russia). The repetitive music exposure was performed using the sequence of: 60 s – music on and then 60 s – music off during 2 h. The sound level was measured directly in a cage of animals using the sound level meter (Megeon 92130, Russia).

***In vivo*****real time confocal microscopy of extravasation of Evans Blue** from the cerebral vessels into brain tissues was performed via the optical window using the adapted protocol for two-photonic imaging of the cortical vessels in awake behavior rodents [79]. Thus, OBBB monitored in unanesthetized rats to avoid the possible effects of anesthesia on the BBB permeability. Ten days before imaging, the chronic optical windows (ø 3 mm) [80] were prepared in coordinates 4 mm lateral to bregma and 3 mm medial on the acrylic platform for EEG electrodes. In parallel, a polyethylene catheter (PE-10 tip, Scientific Commodities Inc., Lake Havasu City, Arizona) was inserted into the right femoral vein for dye intravenous injection in a single bolus dose (2 mg∕100 g, 1% solution in physiological 0.9 % saline). Images were captured with Nikon A1R confocal microscope with dry 10 × 0.45 lens (CFI Plan Apo Lambda S 10X, MRD70100 Nikon Corp., Japan) over 60 min with 1 min interval. At each time point an image stack of 25 slices within 120 µm depth range was captured, then maximum intensity Z projection was calculated.

#### 5.3.1 Ex vivo analysis of OBBB using confocal microscopy

Rats were decapitated and the brains were quickly removed, fixed with 4% neutral buffered formalin for 24 h, and cut into 50-µm thick slices on a vibratome (Leica VT 1000 S Microsystem, Germany). The slices were incubated for one night with the goat anti-mouse NG2 antibody (1:500; ab 50009, Abcam, Cambridge, United Kingdom) and goat anti-rabbit GFAP antibody (1:500; ab 207165, Abcam, Cambridge, United Kingdom). In the next day, after several rinses in phosphate-buffered saline, the slices were incubated for 1 h with 130 µl fluorescent-labeled secondary antibodies (goat anti-mouse IgG (H+L) Alexa Four 647; goat anti-rabbit IgG (H+L) Alexa Four 488; Invitrogen, Molecular Probes, Eugene, Oregon, USA). The slices were analyzed using a confocal microscope (Leica SP5, Germany). Approximately 8–12 slices per animal from cortical and subcortical (excepting hypothalamus and choroid plexus where BBB is leaky) regions were imaged.

#### 5.3.2 Spectrofluorometric assay of EBD extravasation

Evans Blue dye (Sigma Chemical Co., St. Louis, Missouri, 2 mg/ body weight, 1% solution in physiological 0.9 % saline) was injected into the femoral vein and circulated in the blood for 30 min. Then, the rats were decapitated, their brains were quickly collected and placed on ice (no anticoagulation was used during blood collection). Prior to brain removal, the brains were perfused with saline to wash out the remaining dye in the cerebral vessels. The EBD level in brain tissues was evaluated in accordance with the recommended protocol [81] in the IBBB and OBBB groups.

#### 5.3.3 Optical monitoring of FITCD distribution in the brain

Ten days before experiments, we performed implantation of chronic polyethylene catheters into the right lateral ventricle (AP, 1.0 mm; ML, - 1.4 mm; DV, 3.5 mm) for the injection of FITCD (5 µl, at a rate of 0.1 µl/min, 0.5 % solution in saline, Sigma-Aldrich, US) and in the femoral vein according to the protocol reported by Devos et al. [82]. The FITCD was injected into the right lateral ventricle at 11:00 in the wake and OBBB groups (music exposure was started at 08:00, duration of music effects was during 2 h and latency period of OBBB was 1 h, therefore the time of FITCD injection into the ventricle in the OBBB group was 11:00, i.e. 3 h after the start of experiment; to create identical experimental condition, FITCD was injected into the ventricle in the wake group also at 11:00) and at 23:00 in the sleep group, i.e. 3 h after light-off time in the experimental room. After FITCD injection, the cerebral vessels were filled with EBD injected into the tail vein (Sigma Chemical Co., St. Louis, Missouri, 2 mg/ body weight, 1% solution in physiological 0.9% saline) via the implanted polyethylene catheter (PE-10, 0.28 mm ID × 0.61 mm OD, Scientific Commodities Inc., Lake Havasu City, Arizona, United States). The injection of FITCD into the ventricle and EBD in the femoral vein was performed automatically using an automatic syringe injector (QSI, 53311, Stoelting, Wheat Lane, USA).

The *ex vivo* optical study of FITCD distribution from the ventricle in brain tissues and its accumulation in dcLNs was performed 3 h after the intra-ventricular injection of FITCD in rats. Rats were sacrificed, their brains and dcLNs were carefully removed. The imaging was performed using a confocal microscope (Nikon A1R MP, Nikon Instruments Inc.) with a × 20 objective (0.75 NA). Two lasers (488 nm and 560 nm respectively) were used for the excitation of FITCВ and Evans Blue fluorescent dyes respectively during the confocal imaging.

The Petri dishes with samples were submerged in a buffer solution and then were placed on a motorized stage below the objective of A1R MP. The top surface of each sample was covered with a 25 mm × 50 mm× 0.17 mm cover glass. The 12 bit grayscale confocal images were acquired in a dark room. To cover larger regions of interest in the samples each acquired confocal image was stitched out of smaller 512 px by 512 px confocal images with 15% overlap, as the field of view of the × 20 objective was restricted only to 0.5 mm by 0.5 mm area. The images were obtained using NIS-Elements software (Nikon Instruments Inc.) and analyzed using Fiji software (Open-source image processing software). Image processing procedures were identical for each pair of images (control and laser-treated samples) for each channel to ensure an accurate comparison of the fluorescence intensity. The quantitative analysis of tracer in the brain slices was carried out on a fluorescence microscopic system described above. For a quantitative analysis of the intensity signal from FITCD, ImageJ was used for image data processing and analysis. The intensity of fluorescence for each slide was integrated over a rectangular region of interest bounding the brain slice. The integral value was divided by slice area. The areas of brain slices were calculated using the plugin “Analyze Particles” in the “Analyze” tab, which calculates the total area of tracer fluorescence intensity tissue elements—the indicator “Total Area”. In all cases, 10 regions of interest were analyzed.

### 5.4 Analysis of EEG rhythms

We used the spectral analysis for the detection of frequency ranges, which are perspective for further analysis using other techniques. We used the Welch approach to estimate the spectral power density [83], [84], [85]. The signals were divided into 100-second windows with 50-second overlap. To reduce the leakage effect, we used the Bartlett window function. For each window, we calculated periodograms, which were averaged in the frequency domain using the Daniell method [83], [85] with 0.03 Hz averaging windows. The Daniell method was used to suppress the fluctuations caused by noises of various origins.

We also investigated the data, using several techniques of coupling detection. We compared the results to confirm their reliability. We calculated the coherence function C(f).

between the pairs of the EEG signals [83]:

$$C\left(f\right)=\frac{\left|G_{xy}\left(f\right)\right|}{\sqrt{G_{xx}\left(f\right)G_{yy}\left(f\right)}},$$

where $G_{xx}\left(f\right)$and $G_{yy}\left(f\right)$ are the power spectra of the EEG signals and $G_{xy}\left(f\right)$ is a cross spectrum:

$$G_{xy}\left(f\right)=\langle F_{x}\left(f\right)F_{y}^{*}\left(f\right)\rangle ,$$

where $F_{x}\left(f\right)$ and $F_{y}\left(f\right)$ are the Fourier transformations of the EEG signals, the asterisk denotes complex conjugation, and angular brackets denote average over time. The coherence function takes the values between zero and unity and characterizes the phase coherence between oscillations for the frequency *f*. It is considered to be a linear measure of coupling. To estimate the C(f), the EEG signals were divided into 100-second windows with 50-second overlap. The results for each window were then averaged using the Daniell method with 0.13 Hz averaging windows.

To analyze the collective dynamics of EEG signals, we used cross recurrence analysis (CRA) – a well-known method for the analysis of dynamics of complex systems [83], which also shows good results when applied to the heart rate variability signals [85], [86]. CRA is based on the projection of phase portraits of two signals into the same phase space, and the construction of time-domain structures in the form of horizontal and vertical segments, which characterize the proximity of the phase trajectories with a specified degree of accuracy. The obtained diagrams are commonly analyzed qualitatively or used to calculate various quantitative topological measurements. We calculated five indices of CRA. Detailed description is given in the I. Here we only give the maximal length of diagonal lines (*MDL*) that can reflect the degree of nonlinear correlation and the time, which the phase trajectories evolve closely in the phase space. It should be noted that all measures are statistically significant (p < 0.05).

We used the skeleton method of continuous wavelet transformation to assess the oscillation patterns in EEG signal emerging in different frequency ranges [87]. We carried out a preliminary analysis of the number and duration of oscillatory patterns in six frequency ranges (the results are shown in Table 2 of the SI). Further analysis was focused on the low-wave activity range, delta (0–4 Hz) and theta (4–8 Hz).

### 5.5 Analysis of EEG dynamics using an artificial neural network

To automate the recognition of markers arising in the EEG signals, an artificial neural network (ANN) with a direct connection has been created. The input series based on the pure EEG data have not led to useful results. Thus, the characteristic used to produce these input series has been chosen according to the best efficiency of ANN. It is a signal-to-noise ratio (SNR). If X is some random variable, then SNR is the ratio of its expected value E(X) to square root of corresponding variance or standard deviation $\sigma^{2}\left(X\right)=\mathrm{Var}(X)$:

$$\mathrm{SN}R\left(X\right)=\frac{E\left(X\right)}{\sigma \left(X\right)},$$

where

$$E\left(X\right)=\langle X\rangle =\frac{1}{N}\sum_{i=1}^{N}X_{i},$$

$$\sigma \left(X\right)=\frac{1}{N-1}\sum_{i=1}^{N}{\left(X_{i}-\langle X\rangle \right)}^{2}.$$

SNR allows one to show the balance between the level of useful signal and the deviation of this signal from the mean. For given EEG signals, the SNR can be calculated as follows. The time realization is divided into windows of a certain size (here it is 60 s). The original realization step is 0.0005 s, so there are 120,000 points inside each window. The selected window is applied to the realization in each 1 s step for a smoother transition between the obtained results.

ANN consists of an input layer, which has 90 neurons. Each of the input neuron takes one SNR value as an input. Since the time step of the SNR is 1 s, the network can process the window lasting 1.5 min in one pass. There are four hidden layers in ANN. They consist of 500, 50, 500, and 50 neurons, respectively. The output layer has only one neuron, which produces a real number in the range [0; 1]. This number characterizes how far ANN considers the input data similar to those on which it was trained. 1 corresponds to maximum similarity and 0 corresponds to lack of similarity. The activation function of neurons in hidden layers is a sigmoid function $f\left(x\right)=\frac{1}{1+e^{x}}$. The output and input neurons are linear. The network was built and trained using Keras (open neural network library developed for Python) [88].

ANN was trained using EEG signals before and after the stimulus. The EEG time series after the stimulus were marked as the data with opened BBB (their amount for the *i*th animal is denoted as $N_{i}^{s}$), while the rest were noted as a closed one (in the amount of $N_{i}^{d}$). The training data was obtained from EEG of the 1st rat, and then the trained ANN was applied to others. Then the training data was taken from EEG of the 2nd rat and etc. Based on these data, it is possible to calculate the partof data similar to the data from the training set, according to the formula $C^{s}=\frac{\sum_{i}N_{i}^{s}}{\sum_{i}N_{i}^{d}}$.

## Statistical analysis

The analysis of statistical significance using the generation of surrogate data was adopted to test the results of both correlation analysis and detection of directional coupling. We used Amplitude Adjusted Fourier Transform (AAFT) surrogate data, as proposed in [89], to test the statistical hypothesis that the systems are not linearly coupled. The surrogate data were calculated from the original signals and preserved the periodograms but shuffled the phases, destroying correlations between the phases. The results were considered significant at p < 0.05.

## CRediT authorship contribution statement

Writing-original draft, **Oxana Semyachkina-Glushkovslaya, Karavaev A.S., Prokhorov M.D., Runnova A.E., Semenova N.I**. Writing review & editing, **Oxana Semyachkina-Glushkovslaya, Kurths J.G. Visualization, Fedosov I.V., Shirokov A.A., Dubrovski A.I.** Conceptualization, **Oxana Semyachkina-Glushkovslaya, Postnov D.E., Penzel T.U., Kurths J.G.** Project lead, **Oxana Semyachkina-Glushkovslaya, Kurths J.G.** Investigation, **Blokhina I.A., Dubrovski A.I., Terskov A.V., Khorovodov A.P., Ageev V.B., Elovenko D.A., Evsukova A.S., Adushkina V.V., Telnova V.V.** Methodology, **Blokhina I.A., Ageev V.B.** Formal analysis, **Prokhorov M.D., Borovkova E.I., Ishbulatov Yu.M., Hramkov A.N., Kulminskiy D.D., Semenova N.I., Sergeev K.S., Slepnev A.V., Sitnikova E.Yu., Zhuravlev M.O.**

## Competing interests

The authors declare that they have no competing interests.
