# Supplementary material for: EEG biomarkers of activation of the lymphatic drainage system of the brain during sleep and opening of the blood-brain barrier
Source: Comput Struct Biotechnol J. 2022 Dec 15;21:758–68. doi: 10.1016/j.csbj.2022.12.019 (PMC9841170; doi:10.1016/j.csbj.2022.12.019)
Supplement: Supplementary file 1 — Supplementary material [file mmc1.docx]

Supplementary Information for the paper

**EEG biomarkers of activation of the lymphatic drainage system of the brain during sleep and opening of the blood-brain barrier: an innovative strategy in prognosis of brain diseases**

Semyachkina-Glushkovskaya O.V.^1,2^,, Karavaev A.S.^2,3,4^, Prokhorov M.D.^2,3^, Runnova A.E.^2,5^, Borovkova E.I.^2,3,4^, Ishbulatov Yu.M.^2,3,4^, Hramkov A.N.^2^, Kulminskiy D.D.^2,4^, Semenova N.I.^2,6^, Sergeev K.S.^2^, Slepnev A.V.^2^, Sitnikova E.Yu.,^2,7^, Zhuravlev M.O.^2,5^, Fedosov I.V.^2^, Shirokov A.A.^2,7^, Blokhina I.A.^2^, Dubrovski A.I.^2^, Terskov A.V.^2^, Khorovodov A.P.^2^, Ageev V.B.^2^, Elovenko D.A.^2^, Evsukova A.S.^2^, Adushkina V.V.^2^, Telnova V.V.^2^, Postnov D.E.^2^, Penzel T.U.^2,3^, Kurths J.G.^1,2,10^

^1^Physics Department, Humboldt University, Newtonstrasse 15, 12489 Berlin, Germany

^2^Saratov State University, Astrakhanskaya str., 83, Saratov, 410012, Russia

^3^Charité – Universitätsmedizin Berlin, Charitéplatz 1, 10117 Berlin, Germany

^4^Saratov Branch of the Institute of Radio Engineering and Electronics of Russian Academy of Sciences, Zelyonaya, 38, Saratov, 410019, Russia

^5^Saratov State Medical University, B. Kazachaya str., 112, Saratov, 410012, Russia

^6^FEMTO-ST Institut, Université Bourgogne Franche-Comté 15B avenue des Montboucons Besançon Cedex, 25030, France

^7^Institute of Higher Nervous Activity and Neurophysiology of Russian Academy of Sciences, (IHNA&NPh RAS), 5A Butlerova St., Moscow 117485, Russia

^8^Institute of Biochemistry and Physiology of Plants and Microorganisms, Russian Academy of Sciences, Prospekt

Entuziastov 13, Saratov 410049, Russia

^9^Interdisciplinary Center of Sleep Medicine, Berlin, Charité – Universitätsmedizin Berlin, Charitéplatz 1, 10117 Berlin, Germany

^10^Potsdam Institute for Climate Impact Research, Telegrafenberg A31, 14473 Potsdam, Germany

**1. Data analysis**

To supplement and increase the reliability of the results presented in the article, the сross recurrence analysis of the experimental data is considered in detail below, the values of several additionally calculated indices are given. The results of the analysis of directional coupling between the EEG leads obtained using a sensitive method based on modeling the dynamics of instantaneous phases of experimental signals in various frequency bands are also presented.

**1.1. Cross recurrence analysis**

To analyze the collective dynamics of EEG signals, we used the cross recurrence analysis (CRA) that is a well-known method for the analysis of dynamics of complex systems. First, the EEG signals were filtered using band-pass filter to extract the oscillations in the δ- [0-4 Hz] and θ- [4-8 Hz] frequency ranges. Then, the phase space was reconstructed. In accordance with Takens recommendations, we used the delay method and reconstructed the phase space with *D* dimensions and the following axes: $\left\{ x\left( t \right), x\left( t-\tau_{0} \right), \ldots, x\left( t-{{(D}_{0}-1)\tau}_{0} \right) \right\}$. We used the time lag $\tau_{0}$, corresponding to the minimum of the autocorrelation function. For the experimental data, the estimations of $\tau_{0}$ were: 3.3±0.05 s for the wake group, 3.1±0.06 s for the OBBB group, and 2.9±0.03 s for the sleep group. Since the estimations were close for different states, we used the same average delay time $\tau_{0}$=3.1 s to reconstruct the phase spaces in all cases. The number of dimensions of the phase space was estimated using the false neighbor method. In Figure 1, we plotted the functions of probability of false neighbor appearance from the number of dimensions of the phase space. We considered *D* to be sufficient when the probability of false neighbor appearance was smaller than 0.01. From Figure 1(a), it can be seen that all functions reach this level, but at different rates. In δ- frequency ranges during the normal sleep, the estimated *D* was 20, in the awake state it was 25, and in the awake state with the opened BBB it was 35. In θ-frequency range, *D* was 25 for all states (Figure 1(b)). For further analysis, we chose the highest value $D$=45 and used it to calculate the cross recurrence plots (CRP) for each rat in the each state and each frequency range.


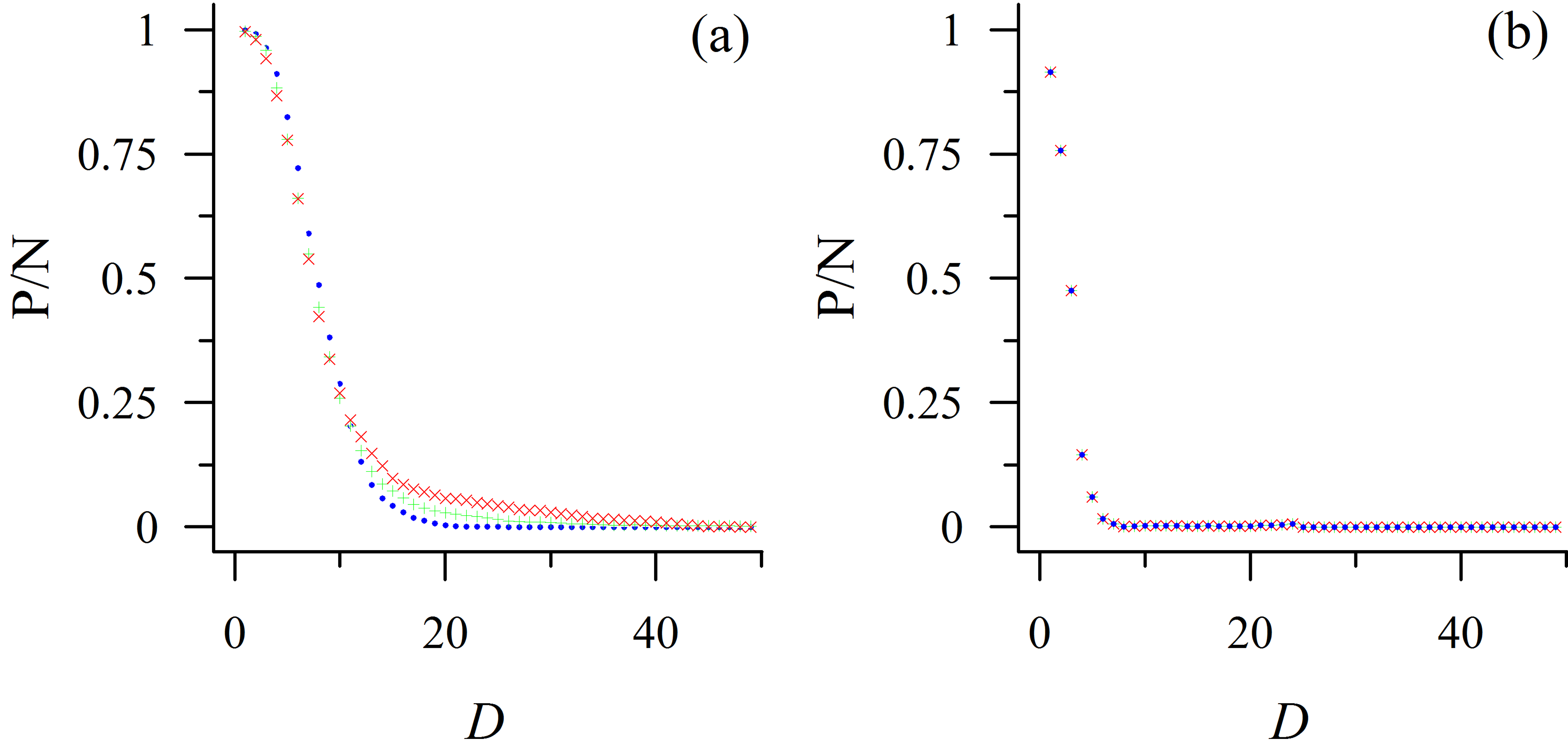


**Fig. 1 Probability of false neighbor detection, calculated separately for different states**. Green pluses correspond to the wake group, blue dots - to the sleep group, and red crosses to the OBBB group. (a) δ-frequency range and (b) θ-frequency range.

The CRP is a visual representation of the closeness of two-phase trajectories in the same phase space. If $\vec{x}_{i}$, $i=1,...,N$, are the points of the first phase trajectory, and $\vec{y}_{i}$ are the points of the second phase trajectory, then the CRP is a $N\times N$ plot, which is calculated as follows:

$CRP_{i,j}=\Theta\left(\varepsilon-\left\|\vec{x}_{i}-\vec{y}_{j}\right\|\right),$ (1)

where $\Theta$ is the Heaviside function, ε is a small value, and $\left\|\vec{x}_{i}-\vec{y}_{j}\right\|$ is the Euclidian distance between the points of the two phase trajectories. Therefore, in the CRP, only those points are non-zero (or so called recurrence points) for which the points of the two phase trajectories are closer than ε. The examples of the CRP are shown in Figure  2. The three top panels (Figure 2(a)) are the CRP plots between the δ-range EEG oscillations in the wake, OBBB, and sleep groups. The three bottom panels (Figure  2(b)) are the CRP plots between the θ−range EEG oscillations measured in the same states.


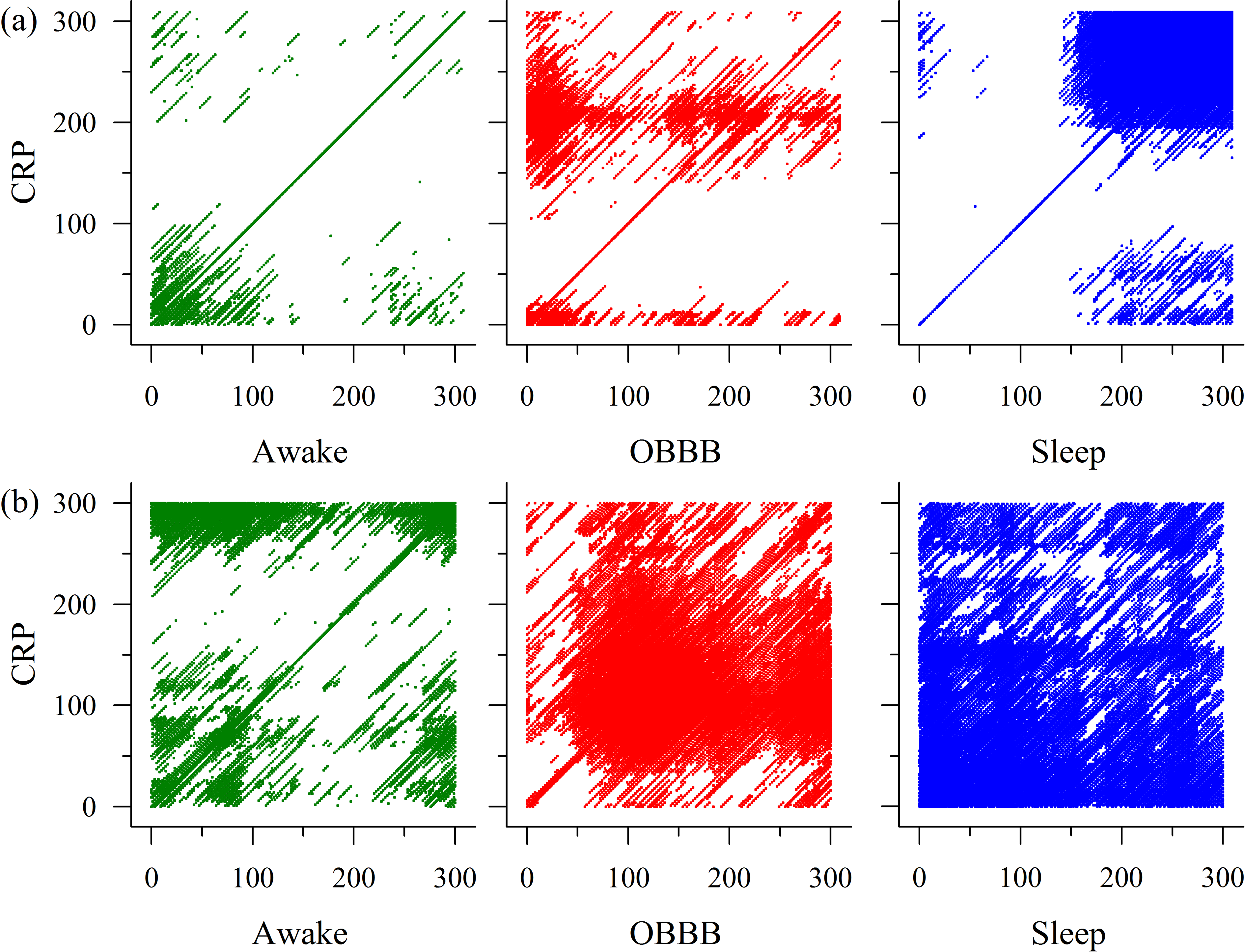


**Fig. 2 Cross recurrence plots** in δ- (a) and θ- (b) frequency ranges, calculated from EEG signals of rat #1. The awake state is shown in green, sleep – in blue, and OBBB – in red.

From Figure 2, it is evident that for the θ-range oscillations, the CRPs contain more recurrence points. For each state, the CRPs are also qualitatively different. However, no quantitative information can be obtained from visual analysis of the CRPs. Therefore, we also calculated a number of well-established numerical indexes from the CRPs. All calculations were performed in accordance with [3].

The recurrence rate index (*RR*) reflects the density of recurrence points in the CRP and is defined as follows:

$RR=\frac{1}{N^{2}}\sum_{i,j=1}^{N} CRP_{i,j}.$ (2)

Next four indexes require the calculation of the histogram of lengths *P*(*l*) for the diagonal lines in the CRP, where *l* is the length of a diagonal line:

$P(l)=\sum_{i,j=1}^{N} \left(1-CRP_{i-1,j-1}\right)\left(1-CRP_{i+l,j+l}\right)\times\prod_{k=0}^{l-1} CRP_{i+k,j+k}.$ (3)

From the *P*(*l*), we calculated the determinism index (*DET*), which is defined as a ratio of all points that form diagonal lines (the minimal lengths is 2 points) to all recurrence points:

$DET=\frac{\sum_{l=2}^{N} lP(l)}{\sum_{l=1}^{N} lP(l)}.$ (4)

The *MDL* index is the maximum length among the diagonal lines:

(5)

where *N_l_* is the total number of the diagonal lines.

Similarly to (3), we calculated the histogram *P*(*v*) for the vertical lines on the CRP:

$P(v)=\sum_{i,j=1}^{N} \left(1-CRP_{i-1,j-1}\right)\left(1-CRP_{i,j+v}\right)\times\prod_{k=0}^{v-1} CRP_{i,j+k}.$ (6)

The laminarity index *(LAM*) is the equivalent of the *DET* index, but for the vertical lines:

$LAM=\frac{\sum_{v=2}^{N} vP(v)}{\sum_{v=1}^{N} vP(v)}.$ (7)

The *MVL* index is the maximum length among the vertical lines:

(8)

where *N_v_* is the total number of the vertical lines.

We calculated the *RR*, *DET*, *MDL*, *LAM*, and *MVL* indexes from the CRP plots, shown in Figure 3. From Figure 3, it is evident that all indexes exhibit similar relations between the wake, OBBB and sleep groups. All indexes show close values for OBBB and sleep.





**Fig. 3 Cross recurrence analysis measures**: (a) ‑ *RR*, (b) ‑ *DET*, (c) ‑ *LAM*, and (d) ‑ *MVL* calculated for each state and averaged over the statistical ensemble with plotted standard error of the mean in δ- (a) and θ- (b) frequency ranges.

**1.2. Phase coupling analysis**

To better distinguish the EEG topology, which is the typical for opened BBB, in further step we analyzed the nonlinear interaction between two EEG channels using the technique of phase coupling detection.

To extract the phases of complex irregular EEG signals, we filter them around the frequencies corresponding to the maximum power of the spectra in δ-band (band-pass filter 0.5-1.0 Hz) and θ-band (band-pass filter 6.5-7.5 Hz) [4, 5]. Then, we calculated the instantaneous phases $\phi_{x}(t)$ and $\phi_{y}(t)$ for these rhythms using the Hilbert transform [6, 7]. The dynamics of the phase signal was described using the first-order phase oscillators [8].

$d\phi_{x}(t)/dt=F_{xy}(\phi_{x}(t),\phi_{y}(t-\Delta))+\xi_{x}(t),$ (9)

where $F_{xy}$ function defines the coupling between the EEG channels *x* and *y*, Δ ‑ delay between the systems. Than we used differential equations to model the phase increment over the period of τ seconds:

$d\phi_{x}(t)=\phi_{x}(t+\tau)-\phi_{x}(t)=F_{xy}(\phi_{x}(t),\phi_{y}(t-\Delta))+\varepsilon_{x}(t),$ (10)

where is Gaussian zero-mean noise, which autocorrelation function drops to zero after $\tau$ seconds, and $F_{xy}$ is the trigonometric polynomial functions. From time series we estimated the coefficients in polynomial functions and calculated the coupling strengths in direction from *y* to *x* for a trial time delay of  Δ:

$G_{xy}(\Delta)\approx\int_{0}^{2\pi} \int_{0}^{2\pi} (\partial F_{xy}(\phi_{x}(t),\phi_{y}(t-\Delta))/\partial\phi_{y})d\phi_{x}d\phi_{y}.$ (11)

We used $\tau$ equal to one characteristic oscillation period in the considered frequency range. The trial delay Δ was varied from 0 to 2.5 s. The same parameters were used when calculating the coupling strengths in the opposite direction $G_{yx}(\Delta)$. The coupling coefficient is normalized to the variance of the instantaneous phase signal of the acting system. Thus, for example, the values of $G_{yx}(\Delta)$ characterize what fraction of the variance of the signal phase x can be described using the values of the signal phase y.

To check the statistical significance of the directional coupling we have used the analytical formula proposed in [9]. The formula allows one to evaluate the significance of the difference between the value of $G_{yx}$ and zero.

Figure 4 shows typical examples of directional phase coupling coefficients. In each frequency band and in each stage, the maximum values of the coupling coefficients are close.

Using the analytic formula from [9] we have shown that all phase coupling coefficients was statistically significant (p=0.05). So we were able to detect significant coupling between the EEG leads. The coupling coefficients were at maximal values of *G*_xy_ and *G*_yx_ which were obtain for rather stable delay times Δ_max_ of 0.47±0.10 s for θ-band and 0.90±0.15 s for δ-band and (меаn delay±standard deviation). Also the coupling coefficients *G*_xy_(Δ_max_) and *G*_yx_(Δ_max_) demonstrated high symmetry in both directions. The coefficient of variation between the maximum values of coupling coefficients with respect to its mean value was 0.17. Therefore, we assumed the coupling to be symmetrical and averaged the maximum values of coupling coefficients over the statistical ensemble: $G=\left\langle G_{xy}\left( \Delta_{\max} \right)+G_{yx}\left( \Delta_{\max} \right) \right\rangle$, where means averaging over ensemble (Figure 4).

Figure 5 shows that in the θ-band, the phase coupling technique distinguishes the open BBB stage from the awake state (0.021±0.001 (OBBB) vs. 0.032±0.003 (awake state)). At the same time, in this band the properties of the phase coupling for the open BBB and sleep stage are close on average (0.021±0.001 (OBBB) vs. 0.021±0.005 (sleep). In the δ-band, the coupling strength between the phases of the EEG leads increases (0.082±0.003 (OBBB), 0.086±0.003 (sleep) and 0.087±0.003 (awake state)). However, the sensitivity of the method is not enough to distinguish between different stages. The values of the phase coupling coefficients in the θ-bandare smaller than in the δ-band for all rats.

**
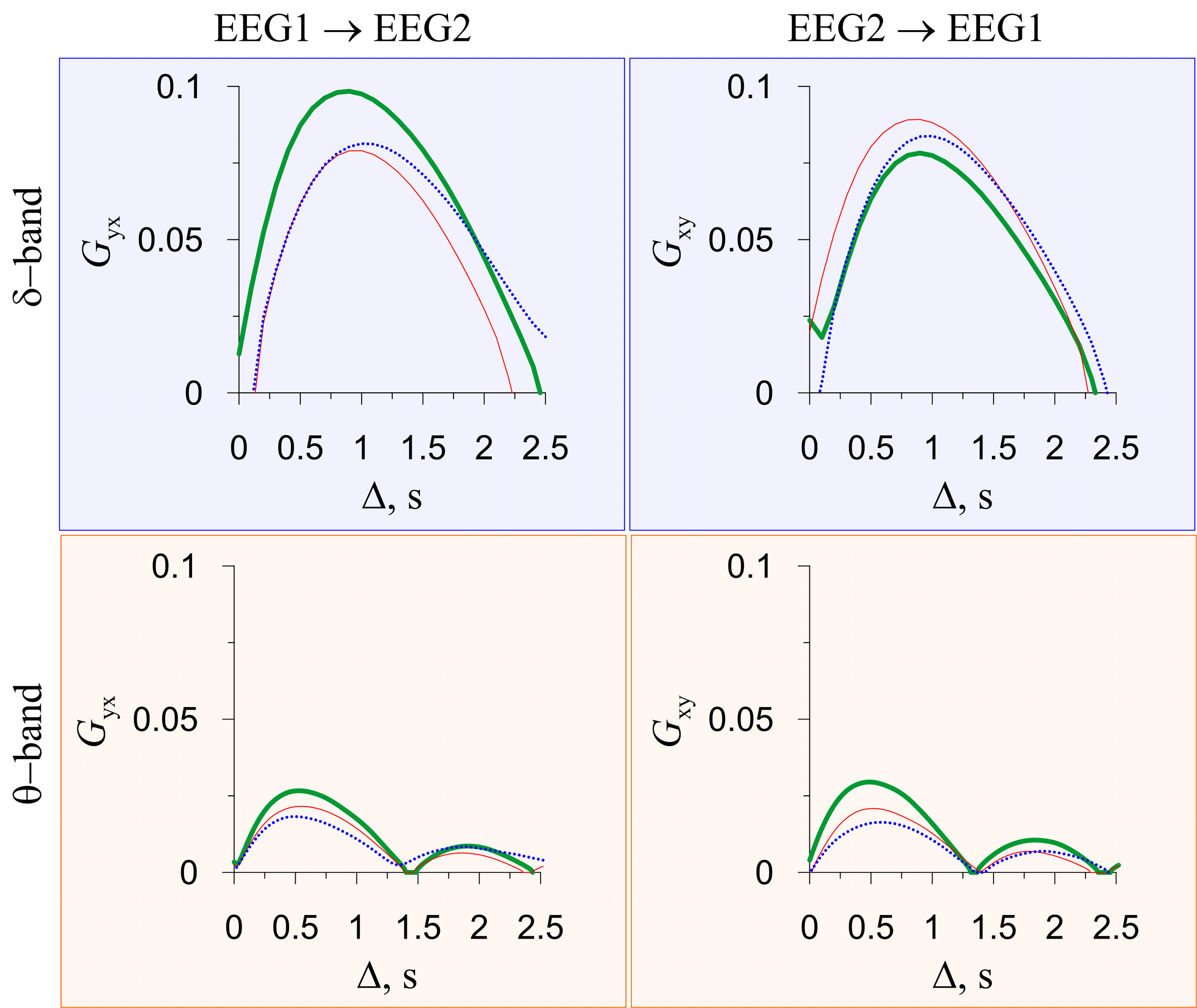
**

**Fig. 4 Directional phase coupling coefficients calculated in δ- and θ-bands**. Bold line corresponds to the awake state, thin line – to OBBB, and dot line – to sleep.


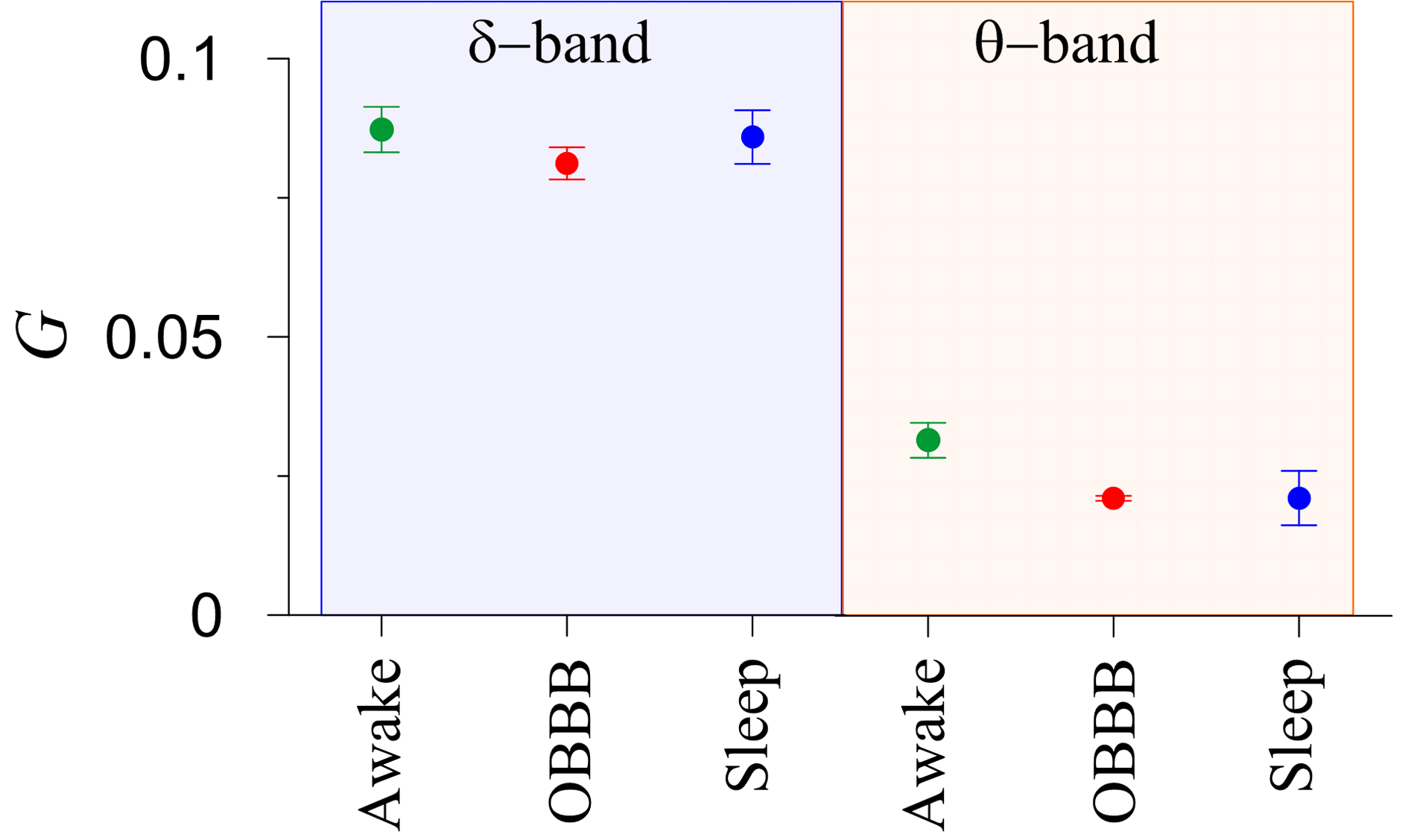


**Fig. 5 Application of phase dynamic modeling to detection the directional coupling between the rat EEG signals** that were registered during sleep, OBBB and in the awake state. Depicted results are typical and we obtained from the rat #1. The coupling coefficients G calculated for each state and averaged over the statistical ensemble with plotted standard error of the mean.

**1.3. Time-frequency wavelet-analysis**

The mathematical basis of the time-frequency analysis is the continuous wavelet transform (CWT) [10, 11]:

$\mathrm{W}(s,t)=\sqrt{s}\int_{-\infty}^{\infty} x(\mathrm{t})\psi^{*}\left(\frac{t-t_{0}}{s}\right)dt,$ (12)

where *x*(*t*) is the EEG signal, *s* is the time scale that determines the wavelet width, “***” is the complex conjugation, and $\psi_{t_{0},s}(\mathrm{t})$ is the complex function basis of the wavelet transform. We use the Morlet wavelet [20] as the CWT basis function:

$\psi_{f,t_{0}}^{*}(\eta)=\frac{1}{\sqrt[4]{\pi}}\exp(j2\pi\eta)\exp\left( \frac{-\eta^{2}}{2} \right).$ (13)

Wavelet Morlet-basis is widely used in the analysis of biological objects signals, allowing to maintain the optimal ratio between frequency and time resolution achieved with CWT (12), (13). Moreover, the using of basis (13) makes it possible to switch from the unusual characteristics of time scales *s* to the traditional representation of the Fourier transform frequencies *f*, i. e. *f* = 1\*s*. In next steps we use the usual classical frequency representation of signals when calculating CWT.

When the CWT-analysis using, each one-dimensional signal *x*(*t*) allows one to estimate a two-dimensional wavelet surface *W*(*f*, *t*) (12), characterizing the oscillational activity for each frequency *f* at any time *t* for the initial signal *x*(*t*). The main advantages of wavelet analysis are the simplicity of the time–frequency sweep of noisy non-stationary signals and the good speed of numerical processing. Using CWT makes it possible to clearly and accurately trace the dynamics of different frequency components in the signal [12, 13]. To achieve this goal, the CWT skeleton method is used to process experimental signals of test animals [13, 14]. The technique of this math method is based on the detection and tracking of local maximum of signal oscillation energy for frequency range at certain time moment. The following relation determines the instant CWT–energy distribution:

*E* (*f*, *t*) = |*W*(*f*, *t*)|^2^. (14)

Further, at each moment of time *t*_0_, we estimate a set of local maximum of the instantaneous energy *E* (*f*, *t*). We can consider this set as a base or “skeleton” of CWT-surface *W* (*f*, *t*) (12), describing main oscillatory components in signal *x*(*t*). For *t*_0_ we define a set of frequencies *f*_i_ = sc*_i_*, *i* = 1 – 3, corresponded the maximum amplitude of the instantaneous energy *E* (*f*_1_, *t*_0_) (14), *E* (*f*_1_, *t*_0_) = max [*E*(*f*, *t*_0_)]. These frequencies sc*_i_* are called CWT skeletons. For the skeletons sc_2_ and sc_3_ the corresponding value *E* (*f*, *t*) (14) decreases, i. e., *E* (*sc*_1_, *t*_0_) > *E* (*sc*_2_, *t*_0_) > *E* (*sc*_3_, *t*_0_). This CWT-technique application significantly reduces the time of numerical calculations and also simplifies further numerical analysis of the time – frequency domains, because this approach does not give a large part of the unnecessary detail of information in CWT-surfaces. In our work, we limite to considering the first three CWT - skeletons, as was done, for example, in [15].

We divide the entire main frequency range of EEG signal registration into six frequency ranges: Δ*f*_1_ [0; 2] Hz, Δ*f*_2_ [2; 4] Hz, Δ*f*_3_ [4; 6] Hz, Δ*f*_4_ [6; 8] Hz, Δ*f*_5_ [8; 12] Hz, Δ*f*_6_ [12; 14] Hz. The frequency ranges Δ*f*_1_ and Δ*f*_2_ are included in the *δ* - band, Δ*f*_3_ and Δ*f*_4_ refer to *θ* - band.

After detection of the entire set of skeletons {*sc*_1_ (*t*), *sc*_2_ (*t*), *sc*_3_ (*t*)} we estimate their distribution on the each of Δ*f*_1-6_ frequencies bands. We analyze a signals *x*(*t*), associated with different physiological states (sleep, awake and opened BB state) and calculate the number *N* of skeletons *sc*, located in each frequency range, i. e. if *sc*_i_ (*t*)∈ Δ*f_k_,* then skeletons number *N_k_* in *k* - frequency range is increased by 1, *N_k_*++.

Next, we estimate the duration and continuity of oscillatory activity patterns in each Δ*f_k_* band. To do this, we consider the following condition on each time interval [*t_n_;* *t_n+1_*] for each frequency *sc*:

|*sc*(*t_n_*) *– sc*(*t_n+1_*)| < *ε*, (15)

where *sc*(*t*) is one of skeletons set {*sc*_1_ (*t*), *sc*_2_ (*t*), *sc*_3_ (*t*)} in certain time moment, *ε* is a numerical constant chosen from the features of experimental signals, *ε* = 0.05, (*t_n+1_* – *t_n_*) = 0,02 c. We suppose that the pattern develops continuously as long as condition (15) is satisfied. Based on estimation of relation (15) we detect the moments of the pattern beginning *t^b^* and the end *t^e^* and compute the pattern’s duration as *T* = (*t^e^* *–* *t_b_*) c.

For each frequency range on signal fragments for each state of the animal, we calculate the number and the duration of patterns. In the experimental group of test animals, we estimate the average for all EEG-recordings and assess the mean and median values, standard deviations. The results are presented in Table 1.

**Table 1 - The results of oscillational patterns estimation in frequency ranges**

**in EEG-recordings in a group of test animals**

| Frequency range | State | Number of patterns, *N* | | | | | Duration of patterns, *T* | | | | |
| --- | --- | --- | --- | --- | --- | --- | --- | --- | --- | --- | --- |
|  |  | Mean | Median | Standard Deviation | Min | Max | Mean | Median | Standard Deviation | Min | Max |
| Δ*f*_1_  (0-2 Hz) | Awake | 0.51 | 0.52 | 0.06 | 0.39 | 0.64 | 2.84 | 2.77 | 0.42 | 2.24 | 4.08 |
|  | Sleep | 0.46 | 0.47 | 0.06 | 0.39 | 0.54 | 2.77 | 2.80 | 0.46 | 1.92 | 3.58 |
|  | OBBB | 0.46 | 0.46 | 0.08 | 0.29 | 0.72 | 2.73 | 2.66 | 0.55 | 1.67 | 4.77 |
| Δ*f*_2_  (2 - 4 Hz) | Awake | 0.67 | 0.68 | 0.05 | 0.52 | 0.76 | 1.51 | 1.54 | 0.22 | 0.93 | 1.92 |
|  | Sleep | 0.62 | 0.64 | 0.06 | 0.51 | 0.68 | 1.50 | 1.49 | 0.24 | 1.49 | 1.98 |
|  | OBBB | 0.62 | 0.62 | 0.12 | 0.20 | 0.98 | 1.54 | 1.52 | 0.41 | 0.64 | 3.12 |
| Δ*f*_3_  (4 - 6 Hz) | Awake | 0.64 | 0.64 | 0.06 | 0.52 | 0.73 | 1.04 | 1.02 | 0.16 | 0.75 | 1.42 |
|  | Sleep | 0.59 | 0.55 | 0.04 | 0.55 | 0.65 | 0.98 | 0.98 | 0.22 | 0.56 | 1.40 |
|  | OBBB | 0.58 | 0.58 | 0.08 | 0.39 | 0.78 | 0.96 | 1.03 | 0.27 | 0.30 | 2.07 |
| Δ*f_4_*  (6 - 8 Hz) | Awake | 1.07 | 1.09 | 0.07 | 0.89 | 1.19 | 1.12 | 1.10 | 0.11 | 0.97 | 1.40 |
|  | Sleep | 1.00 | 1.06 | 0.15 | 0.63 | 1.14 | 1.15 | 1.12 | 0.23 | 1.12 | 1.92 |
|  | OBBB | 0.86 | 0.88 | 0.14 | 0.49 | 1.18 | 1.29 | 1.22 | 0.31 | 0.80 | 2.39 |
| Δ*f*_5_  (8 – 12 Hz) | Awake | 0.95 | 0.94 | 0.10 | 0.75 | 1.31 | 0.54 | 0.54 | 0.06 | 0.41 | 0.62 |
|  | Sleep | 0.88 | 0.86 | 0.13 | 0.64 | 1.15 | 0.53 | 0.53 | 0.07 | 0.40 | 0.71 |
|  | OBBB | 0.76 | 0.78 | 0.17 | 0.20 | 1.13 | 0.51 | 0.52 | 0.10 | 0.30 | 0.72 |
| Δ*f*_6_  (12 – 14 Hz) | Awake | 0.70 | 0.71 | 0.07 | 0.52 | 0.83 | 0.41 | 0.40 | 0.06 | 0.29 | 0.58 |
|  | Sleep | 0.68 | 0.67 | 0.10 | 0.45 | 0.89 | 0.45 | 0.42 | 0.11 | 0.42 | 0.70 |
|  | OBBB | 0.60 | 0.61 | 0.10 | 0.39 | 0.85 | 0.45 | 0.42 | 0.16 | 0.19 | 0.95 |

Based on the data in Table 1, we observe the significant differences of oscillatory activity in EEG, recorded during natural physiological states (sleep and awake states, p <0.05) in low-frequency ranges (Δ*f*_1_ – Δ*f*_4_). In the frequency ranges Δ*f*_5_ and Δ*f*_6_, the statistical differences between these states are unreliable. Further analysis in the main text of the Paper is carried out for the *δ* (Δ*f*_1_ + Δ*f*_2_) and *θ* (Δ*f*_1_ + Δ*f*_2_) frequency ranges of oscillatory activity.

**3. References**

1. Semyachkina-Glushkovskaya, О. et al. Music/sound opens the blood-brain barrier: a readily available approach to brain drug delivery and therapy of brain diseases. *Proceeding in the Royal Society B. (2020) 287: 20202337.*
2. Albus, U. Guide for the care and use of laboratory animals (8th edn). *Laboratory Animals* **46,** 267–268 (2012).
3. González-Gómez, G. H., Infante, O., Martínez-García, P. & Lerma, C. Analysis of diagonals in cross recurrence plots between heart rate and systolic blood pressure during supine position and active standing in healthy adults. *Chaos* **28,** 085704 (2018).
4. Rosenblum, M. G. & Pikovsky, A. S. Detecting direction of coupling in interacting oscillators. *Phys. Rev. E.* **64,** 045202(R) (2001).
5. Smirnov, D. A. & Bezruchko B. P. Estimation of interaction strength and direction from short and noisy time series. *Phys. Rev. E.* **68,** 059901 (2003).
6. Pikovsky, A. S., Rosenblum, M. G., Osipov, G. V. & Kurths, J. Phase synchronization of chaotic oscillators by external driving. *Physica D* **104,** 219–238 (1997).
7. Gabor, D. Theory of communication. Part 1: The analysis of information. *Journal of the Institution of Electrical Engineers* **93,** 429–441 (1946).
8. Kuramoto, Y. *Chemical Oscillations, Waves, and Turbulence* (Springer, Berlin, 1984).
9. Smirnov, D. A. & Bezruchko, B. P. Detection of couplings in ensembles of stochastic oscillators. *Phys. Rev. E.* **79,** 046204 (2009).
10. Torresani, B. *Continuous Wavelet Transform* (Savoire, Paris, 1995).
11. Hramov, A. E. et al. *Wavelets in Neuroscience* (Springer, Berlin, 2015).
12. Makarov, V. V. et al. Betweenness centrality in multiplex brain network during mental task evaluation. *Phys. Rev. E.* **98,** 062413 (2018).
13. Sitnikova, E. Hramov, A. E., Grubov, V. & Koronovsky, A. A. Age-dependent increase of absence seizures and intrinsic frequency dynamics of sleep spindles in rats. *Neuroscience journal* **2014,** 370764 (2014).
14. Sitnikova, E., Hramov, A. E., Grubov, V. & Koronovsky, A. A. Time-frequency characteristics and dynamics of sleep spindles in WAG/RIJ rats with absence epilepsy. *Brain research* **1543,** 290–299 (2014).
15. Maksimenko, V. A. et al. Visual perception affected by motivation and alertness controlled by a noninvasive brain-computer interface. *PloS ONE* **12,** e0188700 (2017).
